# The germinal center antibody response in health and disease

**DOI:** 10.12688/f1000research.7717.1

**Published:** 2016-05-25

**Authors:** Anthony L. DeFranco

**Affiliations:** 1 University of California, San Francisco, CA, USA

**Keywords:** germinal center, germinal center response, somatic hypermutation, innate immunity

## Abstract

The germinal center response is the delayed but sustained phase of the antibody response that is responsible for producing high-affinity antibodies of the IgG, IgA and/or IgE isotypes. B cells in the germinal center undergo re-iterative cycles of somatic hypermutation of immunoglobulin gene variable regions, clonal expansion, and Darwinian selection for cells expressing higher-affinity antibody variants. Alternatively, selected B cells can terminally differentiate into long-lived plasma cells or into a broad diversity of mutated memory B cells; the former secrete the improved antibodies to fight an infection and to provide continuing protection from re-infection, whereas the latter may jumpstart immune responses to subsequent infections with related but distinct infecting agents. Our understanding of the molecules involved in the germinal center reaction has been informed by studies of human immunodeficiency patients with selective defects in the production of antibodies. Recent studies have begun to reveal how innate immune recognition via Toll-like receptors can enhance the magnitude and selective properties of the germinal center, leading to more effective control of infection by a subset of viruses. Just as early insights into the nature of the germinal center found application in the development of the highly successful conjugate vaccines, more recent insights may find application in the current efforts to develop new generations of vaccines, including vaccines that can induce broadly protective neutralizing antibodies against influenza virus or HIV-1.

## Introduction

The germinal center (GC) reaction is a critical part of the antibody response in which antigen-specific B lymphocytes undergo a sustained period of rapid cellular proliferation, high-level mutagenesis of their antibody genes, and stringent Darwinian selection for those B cells within the GC that make higher-affinity antibodies. The output of the GC is both memory B cells and antibody-secreting cells (called “plasma cells”), and the affinity of both increases for weeks to months, as long as the GC persists, resulting in “affinity maturation” of the antibodies in the blood ^1^. At the same time, there is a change in the type of antibodies being produced, from IgM to IgG, IgA, or IgE, which have more specialized properties with respect to their localization and effector functions.

While somatic mutation and antibody class switch occur to some extent outside of GCs, these processes occur to a much greater extent in the GC. Moreover, whereas the plasma cells generated prior to the GC are almost all programmed to die after a few days, many of the plasma cells generated from the GC can migrate to the bone marrow, where they access survival niches that allow them to live for years ^1, 2^. These long-lived plasma cells are responsible for more than half of the IgG in blood and provide a continual protection against re-infection with viruses and microbes. IgA and IgE have somewhat distinct biology, but nonetheless these isotypes also may provide a more long-lasting protection than the more rapidly turning over IgM.

This review will cover recent progress in understanding the cellular and molecular mechanisms controlling the GC response and recent evidence that innate immune pathways can substantially impact the quality of the GC response. In addition, the importance of the GC response in health and disease will be highlighted. As demonstrated by the susceptibilities of immunodeficient individuals with genetic defects in genes required for the GC response, much of immunity from re-infection with pathogens seen previously and of the success of vaccination can be traced to the GC reaction and its output of long-lived plasma cells, memory B cells, and memory helper T cells. Indeed, an understanding of the basics of T cell-dependent antibody responses leading to GCs was used to develop the improved “conjugate vaccines” against a number of bacterial pathogens, starting in the 1990s ^3^. Efforts to improve current vaccines and to create new vaccines against difficult targets also hope to take advantage of the rapidly increasing understanding of the GC reaction. Development of vaccines that induce more broadly crossreactive neutralizing antibodies against influenza virus ^4, 5^ and HIV-1 ^6^ is an especially active effort at this time. Conversely, the pathogenic autoantibodies made in some autoimmune diseases likely emanate from the GC, so approaches to inhibiting the GC response may have therapeutic value in such patients.

## Early response to antigen and commitment to the germinal center reaction

**Figure 1.**
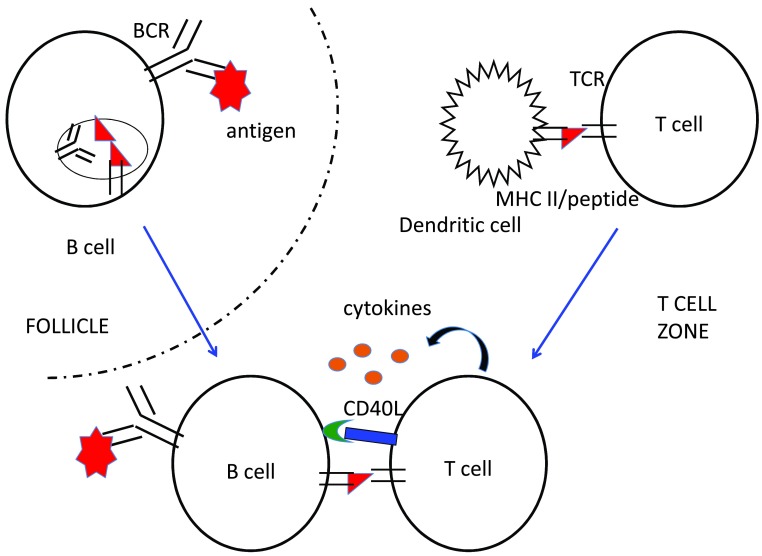
Early events in the antibody response. Antigen-specific B cells contact the incoming antigen (red polygon) in the B cell-rich follicles of secondary lymphoid tissues. Low levels of signaling from the B-cell antigen receptor (BCR) are sufficient to alter responsiveness to chemotactic stimuli such as chemokines, causing the B cell to move out of the follicle into the extrafollicular region adjacent to the T-cell zone. Low-level BCR signaling is also sufficient to promote internalization of the BCR-antigen complex into endocytic compartments, where protein antigens are degraded into peptides (red triangles), which are loaded onto major histocompatibility complex (MHC) class II molecules. Once bound with a peptide, MHC class II molecules traffic to the cell surface, where they can be recognized by the T-cell receptors (TCRs) of T cells that are also specific for the same antigen. Naïve T cells are found in the T-cell zone, where they may become activated by dendritic cells presenting antigenic peptides bound to class II MHC molecules. This activation induces movement of the helper T cell to the boundary of the T-cell zone and the follicle, where they scan B cells for the presence of their TCR ligand. TCR recognition induces prolonged contact with the antigen-presenting B cell and expression of CD40L and of cytokines, which promote activation and clonal expansion of antigen-specific B cells. Interactions between B cells and helper T cells occur approximately 24 hours after antigen arrival in the secondary lymphoid organ and continue in this location until the start of the GC reaction, on day 4 or 5.

Naïve B cells and T cells encounter antigen in separate regions of the secondary lymphoid organ where antigen is first concentrated (spleen, lymph nodes, or Peyer’s patches for antigen in the blood, tissues, and gut lumen, respectively). B cells recognize antigen via the B-cell antigen receptor (BCR), which is a complex between a transmembrane form of the antibody made by that B cell (membrane immunoglobulin), with a heterodimer of two signaling chains called Igα and Igβ. T cells use a similar receptor complex, the T-cell receptor (TCR), containing similar but distinct polypeptides. Rather than recognizing antigen in its native configuration, the TCR recognizes a peptide derived from the antigen bound to the binding groove of one of two types of similar cell surface molecules encoded in the major histocompatibility complex (MHC) and therefore called MHC class I and class II molecules. Antigen encounter by antigen-specific B cells induces changes in expression of receptors for chemotactic cues such as chemokines that direct migration of the cells out of the B cell-rich follicle and to the area between the follicles and the T-cell zone ^7^. Complementary changes are induced in parallel in the antigen-stimulated helper T cells, causing them to migrate to the same place as the antigen-stimulated B cells ^8^. Thus, initial antigen encounter causes antigen-stimulated B cells and helper T cells to relocalize to the same zone within the lymphoid organ and thereby facilitate their interaction ( Figure 1).

B cells also use their BCR to internalize antigen, which is critical for their interactions with helper T cells. The internalized antigen becomes partially degraded in endocytic vesicles and, if it has an attached protein component, the resulting antigen-derived peptides are loaded onto MHC class II molecules, which then traffic to the B-cell surface for recognition by antigen-specific helper T cells ^9^. Recognition by the activated helper T cell of its specific peptide/MHC II complex on the B cell focuses the former’s stimulatory signals on B cells that can recognize an element of the same antigen complex ( Figure 1).

The phase of an antibody response that occurs prior to the GC is referred to as the extrafollicular response. This early phase of the response is characterized by rapid clonal expansion of the antigen-specific B cells and helper T cells, and their interactions contribute importantly to each other’s clonal expansion. During the pre-GC phase, antigen-specific B cells and helper T cells make fate choices between becoming effector cells (plasma cells or effector CD4 T cells) versus migrating into the nascent GC and participating in the slower but higher-quality GC antibody response ^1^. After the first day or so of the response, the helper T cells that have made contact with antigen-specific B cells are recognizable as distinct from effector CD4 T cells and are already referred to as follicular helper T (T _FH_) cells, although they do not actually enter the B cell-rich follicles until the GC response proper is ready to start ^10^. By 4–5 days after antigen encounter, some antigen-specific B cells have terminally differentiated into plasma cells, some have become early memory B cells, and some have upregulated the transcriptional repressor Bcl6 and thereby have committed to the GC response ^11^. The GC-committed cells migrate back into the follicle where they first encountered antigen. Although how cells choose to become memory B cells is poorly understood, it is clear that the choice between GC phenotype and plasma cell phenotype is determined by the competing cellular programs driven by Bcl6 (GC program) and the transcriptional regulator BLIMP-1 (plasma cell program) ^12^. Remarkably, the same two transcriptional regulators drive the corresponding programs in CD4 T cells: BLIMP-1 drives the program of effector CD4 T cells, and Bcl6 drives the T _FH_ cell program, both during the pre-GC phase of the response and also during the GC phase proper ^10^. The effector versus T _FH_ cell fate decision likely occurs soon after antigen encounter and prior to migration to meet up with antigen-stimulated B cells; nonetheless, full attainment of the T _FH_ cell program, including the ability to localize to the GC itself, requires reinforcement by interaction with antigen-presenting B cells in the extrafollicular region.

The GC is first evident histologically at about day 5 of the immune response, and at this point both GC B cells and T _FH_ cells take on characteristic cell surface phenotypes that allow their enumeration and isolation. GC B cells gain expression of Fas, PD-L1, and carbohydrates detected with the lectin peanut agglutinin and with the monoclonal antibody GL-7 and also downregulate IgD. T _FH_ cells further upregulate CXCR5 and PD-1, such that the cells with highest expression of these markers are those CD4 T cells found in the GC, whereas CD4 T cells with somewhat lower levels of these proteins are found in the T-B boundary where interactions with antigen-specific B cells initiate ^10^.

## Mechanism of class switch and somatic mutation

As mentioned above, central to the GC pathway are hypermutation of Ig gene variable regions and class-switch recombination to change the constant regions of the Ig heavy chain. Strongly upregulated in GC B cells is the gene encoding activation-induced cytidine deaminase (AID), an enzyme that converts cytidine in DNA to uridine. In this way, AID initiates somatic hypermutation and class-switch recombination ^13^. AID is expressed at a low level in B cells during the pre-GC phase of the antibody response and is further upregulated in GC B cells, which corresponds to the relative rates of mutation and class switch in these two phases of the antibody response. People and mice with deleterious mutations of the gene encoding AID exhibit strong defects in both somatic mutation and class-switch recombination and correspondingly poor antibody-mediated immunity ^14, 15^. It was initially surprising that the same enzyme was required for both of these events, leading to the hypothesis that the role of AID was indirect ^15^. More recently, however, a strong consensus has emerged that AID is directly responsible both for somatic hypermutation and for class-switch recombination. The enzymology of DNA repair is complex, but the mutagenic effect of AID is enhanced by error-prone DNA repair mechanisms, which can spread mutation to bases near the originally targeted cytidine residue ^13^. Thus, the properties of somatic hypermutation are readily accounted for by direct action of AID combined with various DNA repair mechanisms. Class-switch recombination starts with double-stranded DNA breaks in the “switch” regions adjacent to the exons encoding the various Ig heavy-chain constant regions. Switch regions have a very high density of AID-preferred sequence motifs ^16^, so it is likely that AID deaminates multiple nearby cytidines on both DNA strands, followed by a double-stranded DNA break, which can lead to class-switch recombination ^17, 18^. Although AID can cause mutations in transcribed genes other than Ig genes, its action appears to be concentrated in switch regions and in the variable regions of assembled antibody genes. How this occurs remains an unsolved problem.

## Repeated cycles of somatic hypermutation, proliferation, and selection

The GC reaction first becomes evident after a substantial clonal expansions of the few clonally distinct antigen-specific B cells that are the founders of each GC. Intravital microscopic imagining studies have shown that GC B cells are highly restricted to their GC and do not travel between different GCs in the same lymph node, spleen, or Peyer’s patch or do so infrequently ^19, 20^. This restriction of GC B cells and their clonal progeny to a single GC is important because around day 7, there begins a stringent affinity-based selection within a GC in which the mutated expanded cells within a given GC compete with one another on the basis of their affinities for antigen. Thus, as an immune response has several GCs, each seeded with different B cells at the start of the GC phase of the response, affinity maturation can proceed independently in each GC, likely maintaining a diversity of epitopes recognized by the different GC B cells. It should be noted, however, that entry into an active GC by naïve or recently activated B cells readily occurs ^11^, so it is possible for new competitors to enter a GC after it has started its selective processes and impact the Darwinian selection in the GC. Biologically, antibodies to some epitopes are likely to be more protective than antibodies to other epitopes; for example, antibodies that block virus infection of cells (“neutralization”) typically bind to regions on the virus particle that dock with the target cell. Thus, an important consideration in vaccine design is whether the induced GC response is adequately focused on the desired epitopes ^21^.

**Figure 2.**
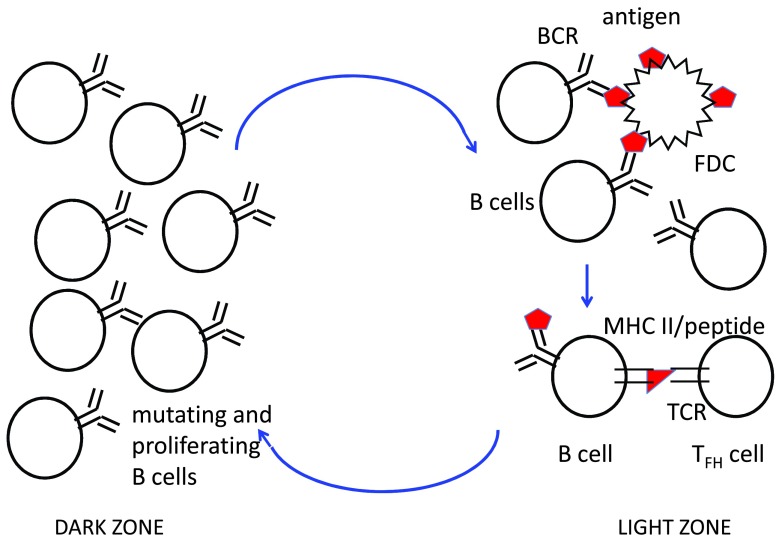
Cyclical movement of B cells between the dark zone and light zone of the germinal center (GC). In the light zone, GC B cells extract antigen (red polygon) from follicular dendritic cells (FDCs) in an affinity-dependent manner, internalize it into endosomes, partially degrade it into peptides (red triangles), and load those peptides onto major histocompatibility complex (MHC) class II molecules as in Figure 1. Follicular helper T (T _FH_) cells rapidly scan the B cells in the light zone and provide stimulatory signals (CD40L and cytokines as in Figure 1) to those B cells that present the most antigen. These signals promote the survival of B cells and also induce c-Myc, promoting proliferation in the dark zone, which is preceded by somatic hypermutation of immunoglobulin gene variable regions by activation-induced cytidine deaminase. After several rounds of proliferation in the dark zone, the mutated clonal progeny migrate back to the light zone and compete for uptake of antigen and receipt of T-cell help. These cycles of mutation, clonal expansion, and selection repeat for weeks to months, as long as the GC response continues. Memory B cells are generated throughout the GC reaction, whereas long-lived plasma cells are preferentially generated from high-affinity GC B cells late in the response. Abbreviations: BCR, B-cell antigen receptor; TCR, T-cell receptor.

At about the same time that affinity selection begins within the GC, the GC can be seen in histological sections to have a “dark” zone, which contains many dividing B cells, and a “light” zone, which contains GC B cells as well as T _FH_ cells and antigen trapped on follicular dendritic cells (FDCs) ( Figure 2). FDCs are a non-hematopoietic stromal cell of specialized function that express complement receptors and Fcγ receptors and use these receptors to trap antigens and hold them on its cell surface. The light zone is where selection based on affinity occurs, and the selected cells migrate to the dark zone to somatically mutate their Ig genes, proliferate, and then migrate back to the light zone for another round of selection ( Figure 2). While proliferating GC B cells are concentrated in the dark zone, accounting for its histological appearance, this distinction is not absolute, and so recently there has been a move away from the earlier nomenclature of referring to dark-zone GC B cells as “centroblasts” and light-zone GC B cells as “centrocytes”. Dark-zone and light-zone GC B cells can be distinguished reasonably well by flow cytometry by using relative changes in cell surface markers, including those that control the migration between the two zones, such as the chemokine receptor CXCR4, which attracts GC B cells to the dark zone and is more highly expressed on dark-zone GC B cells ^11^.

The dark-zone and light-zone subcompartments of the GC appear to promote efficient affinity maturation but are not absolutely required. For example, deletion of the gene encoding CXCR4 in GC B cells prevents migration from the light zone to the dark zone ^22^ and compromises the efficiency of selection for higher-affinity B cells, but some improvement in affinity still occurs. Interestingly, the distinctive properties of dark-zone GC B cells still are observed in a subset of CXCR4 ^−/−^ GC B cells, indicating that cues from the microenvironment of the dark zone are not required to induce the dark-zone phenotype but rather the phenotypic changes are part of a cell-intrinsic program initiated in the light zone ^23^. Interactions of higher-affinity GC B cells with T _FH_ cells in the light zone are thought to promote their survival via the Bcl2-family member Mcl1 ^24^, induce changes in expression of chemoattractant receptors to promote migration back to the dark zone (i.e., CXCR4), and induce expression of the key cell cycle regulator c-Myc ^25, 26^, leading to several rounds of proliferation ^27^.

Early studies of the accumulation of mutations and increased affinity during the antibody response concluded that the GC response involves multiple rounds of mutation, cellular proliferation, and selection, resulting in increased affinity over time, a conclusion supported by recent intravital imaging studies ^11, 28^. As mentioned above, each round of mutation and selection is coupled with migration to the dark zone and back to the light zone. The mechanism of affinity selection in the GC is still an area of active investigation, but the following model is reasonably well supported by experimental data ^11^. Following a short period of somatic mutation by AID and several rounds of cell division in the dark zone, the GC B cell enters the light zone and uses its BCR to extract antigen from the surface of FDCs. It is believed that the affinity of the GC B cell for antigen determines how much antigen it can extract from the FDC (see below). The antigen internalized by a GC B cell is processed into peptides, which are loaded onto MHC class II molecules and trafficked to the cell surface for recognition by T _FH_ cells. The T _FH_ cells actively migrate within the light zone of the GC, where they form relatively short-lived (minutes in duration) associations with antigen-presenting B cells ^19^. Thus, T _FH_ cells are sampling many different GC B cells, and evidently this broad sampling allows the T _FH_ cells to calibrate their response such that they form the longest contacts with those GC B cells expressing the greatest number of cognate peptide/MHC II complexes and thus provide the strongest survival and proliferation signal to the higher-affinity B cells ^27, 29^. BCR signaling induced by antigen may also promote survival of GC B cells, but it is probably not the limiting factor that shapes selection in the GC ^11^. Rather, current evidence indicates that T _FH_ cells provide the key selection signals that maintain GC B-cell survival and allow them to re-enter the dark zone and participate in a new round of mutation and clonal expansion, followed by migration back to the light zone and further selection ( Figure 2).

The selective signals provided by T _FH_ cells to GC B cells are delivered by cytokines, with interleukin-21 (IL-21) being especially important ^30, 31^, and by the cell-bound tumor necrosis factor (TNF)-superfamily member CD40L ^11^. Blocking CD40L in mice with anti-CD40L antibodies at any time during a GC response leads to rapid dissolution of the GC ^11^. Similarly, genetic defects in the X-linked CD40L result in X-linked hyper-IgM syndrome ^32– 34^, in which individuals make IgM but no class-switched isotypes and fail to make GC responses. A very similar clinical syndrome results from genetic deficiency in the autosomally encoded AID ^14^. In addition, GC B cells express inducible T-cell co-stimulator ligand (ICOS-L), the ligand for ICOS, the inducible co-stimulatory molecule. ICOS-L provides important co-stimulation to T _FH_ cells to increase the synthesis of IL-21 and CD40L. In the absence of ICOS, individuals have greatly impaired antibody responses and are included within an immunodeficiency category of diverse genetic causes called combined variable immunodeficiency ^35^, which is similar to hyper-IgM syndrome in that the GC response is largely defective. The interactions of GC B cells and T _FH_ cells also require adhesion molecules of the signaling lymphocytic activation molecule (SLAM) family that signal through the adaptor SLAM-associated protein (SAP), as indicated by defects in the GC response in the genetic immunodeficiency disease X-linked lymphoproliferative syndrome (XLP), caused by loss of function mutations in SAP ^36^. In XLP, the ability of cytotoxic T cells to control the proliferation of Epstein-Barr virus-infected B cells is compromised, leading to the observed lymphoproliferation, but GC responses are also thought to be poor in these individuals ^36^.

Although the competition in the GC is primarily between B cells in the same GC, this process is influenced by the soluble antibody that has been secreted up to that point in the response. The antigen on the surface of FDCs has bound to it secreted antibody ^37^, so the GC B cells must compete with this bound antibody to be able to extract antigen, providing one driver to enhance affinity on an epitope-by-epitope basis and representing a competition between different GCs. The ability of GC B cells to extract antigen from FDC likely involves interactions of the BCR with the cytoskeleton inside the GC B cells, permitting mechanical strength to be generated as part of the process ^38^.

## Immune tolerance to self and the germinal center response

Somatic hypermutation of antibody genes runs the risk of generating variants with increased reactivity to self-antigens ^1^. Indeed, analysis of anti-nuclear antibodies from patients with lupus indicates that their affinity was substantially enhanced by somatic mutations, probably in GC responses ^39, 40^. Similarly, GC responses have been shown to be important for autoantibody production in some, but perhaps not all, mouse models of lupus ^41– 43^. One documented mechanism that purges self-reactive GC B cells is that their expression of Fas makes them susceptible to killing by FasL expressed by helper T cells ^44, 45^, although exactly how that distinguishes self-reactive B cells from B cells responding to the foreign antigen remains unclear. The inhibitory FcγRIIB has also been implicated as important for a B-cell tolerance checkpoint in the re-stimulation of memory B cells ^46^.

As the receipt of help from T _FH_ cells is thought to be the limiting factor in affinity selection, one would expect that GC B cells that have acquired increased specificity for self-antigens would still need to be able to bind and internalize the initiating antigen in order to present peptides to T _FH_ cells and be selected to survive and expand. Nonetheless, it may be that in some cases there is sufficient cross-reactivity between a foreign antigen and a self-antigen that GC B cells with increased affinity for a self-antigen would be selected and become plasma cells secreting the autoantigen. Perhaps the best-documented example of this “molecular mimicry” model for autoimmune disease in humans is in Guillain-Barré syndrome, where some strains of *Campylobacter* make a carbohydrate that is very similar to gangliosides on peripheral neurons, and some of the people who experience a severe infection with the corresponding strains make anti-ganglioside antibodies that likely cause the resulting peripheral neuropathy ^47^. Less is known about the mechanisms by which autoimmune responses are triggered for most other antibody-mediated autoimmune diseases, although as mentioned above there is evidence for the importance of somatic mutations for development of anti-DNA antibodies in lupus. The possible role of Toll-like receptors (TLRs) in this process is discussed below.

## Adjuvants promote the quality of the germinal center response

Both the magnitude of the antibody response and the degree of affinity maturation are strongly influenced by the adjuvants used in a vaccination ^48^. TLRs have emerged as an especially important innate immune pathway for promoting the antibody response. Whereas an early study had indicated that TLR recognition by B cells could promote the antibody response when using a pure TLR ligand as the adjuvant ^49^, another study found that mice doubly deficient for the two main TLR signaling adaptor molecules, MyD88 and TRIF, responded normally to immunization with standard adjuvants ^50^, likely reflecting alternative innate immune pathways also stimulated by such adjuvants. A variety of other studies have clearly demonstrated that TLR ligands make excellent adjuvants ^51^; indeed, one such ligand is currently approved for use in human vaccines ^52^. Indeed, live attenuated viral vaccines are among the best vaccines in human practice ^53^, and virus-like particles, in which nucleic acid ligands for TLR7 or TLR9 are present inside the particle ^54, 55^ or nanoparticles with antigen and TLR ligands attached ^48^, also induce outstanding antibody responses.

Whereas these studies established that TLRs can serve as adjuvants for antibody responses, some initial studies suggested that they did so by promoting a strong extrafollicular antibody response ^56^. Subsequent studies, however, have made it clear that TLR recognition can dramatically enhance the GC response, both in its magnitude and in the degree of affinity maturation ^48, 55, 57^. Ligand recognition by the TLRs of both DCs and GC B cells promotes the GC response, but in different ways ^57^. DC recognition of nucleic acid ligands for TLR7 or TLR9 promotes the magnitude of the GC response and increases the overall amount of IgG specific for the antigen but does not enhance affinity maturation. The effect of recognition by DC TLRs is likely a reflection of stronger generation of T _FH_ cells during the early part of the response. In contrast, GC B-cell recognition of TLR7 or TLR9 ligands has a minimal effect on the total amount of specific IgG produced but substantially enhances the quality of the GC response ^48, 57^, including affinity maturation, the number of memory B cells produced, and the isotype of IgG produced, favoring a more inflammatory isotype of IgG ^57^. The ability of TLR7 or TLR9 in the antigen-specific B cell to enhance the GC response is dependent on the nature of the antigen; monomeric protein antigens, which have a limited ability to induce BCR signaling, poorly engage TLR7 or TLR9 on B cells and have little impact, whereas oligomeric haptenated-protein antigens with TLR ligands attached exhibit a several-fold enhancement in the response, and for highly repetitive virus particles, there is a dramatic positive effect on the GC response, and production of IgG increases up to 30-fold ^55^. In mice, the ability of TLR7 or TLR9 in the B cell to respond to virus genomic material has been found to be critical to the generation of neutralizing antibodies and the ability to control virus infection for acute infection with Friend erythroleukemia virus ^58^, the chronic version of lymphocytic choriomeningitis virus (LCMV clone 13) ^59, 60^, and endogenous murine leukemia viruses ^61^. Thus, it is likely that the enhancement of the GC response by TLR7 or TLR9 action in B cells is an evolutionarily selected mechanism to aid in generation of high-affinity antibodies to defend against viruses. The molecular mechanisms of this enhancement of the GC response are the subject of ongoing efforts.

## Toll-like receptors and autoantibody responses

Although TLR7 and TLR9 in B cells promote immune defense against viruses by permitting efficient generation of neutralizing antibodies, the same pathway has been implicated in the production of pathogenic antibodies in the systemic autoimmune disease, systemic lupus erythematosus ^62^. The ability of the BCR and TLRs to synergize for B-cell activation was first recognized in studies examining B-cell proliferation *in vitro* ^62^. Subsequent studies showed that, in the MRL/ *lpr* mouse model of lupus, TLR7 and TLR9 contributed critically to production of anti-ribonucleoprotein IgG antibodies and to production of anti-double-stranded DNA antibodies, respectively ^63^. Moreover, the TLR signaling component MyD88 was shown to be required in both DCs and in B cells in the *Lyn* ^−/−^ mouse model of lupus ^43^. In this mouse model, the GC response has been implicated, since the autoantibodies are absent if SAP is deleted, which affects primarily the GC response and not the extrafollicular antibody response ^36^. Similarly, in a separate mouse model of lupus-like autoimmunity in which TLR7 expression is increased via a transgene, autoantibody production was dependent on the GC response and on TLR7 action in B cells ^64^. In human lupus, affinity maturation is typically observed for the antibodies that recognize double-stranded DNA ^65^. Noteworthy in this regard, both human and mouse B cells express TLR7 and TLR9. Thus, it appears likely that TLR7 or TLR9 in autoreactive B cells contributes importantly to promoting affinity maturation of autoantibodies, likely enhancing their pathogenicity. Whether this pathway can be targeted therapeutically in patients with lupus remains to be seen, but several companies are currently testing TLR antagonists and chemical inhibitors of IL-1 receptor-associated kinases (IRAKs), which are required signaling molecules recruited by the adaptor MyD88. Also of note in this regard is that often lupus is treated with hydroxychloroquine, which decreases the acidity of late endosomes, although this has not been proven to be effective in a randomized clinical trial. As the processing of TLR9 and TLR7 into their active forms involves acid-requiring proteases in endosomes ^66^, the efficacy of hydroxychloroquine in patients with systemic lupus erythematosus may result- from inhibition of TLR7 and TLR9 activity.

## Summary

The GC response is a critical element of the antibody response that is responsible for the production of high-affinity antibodies, long-lived plasma cells secreting these high-quality antibodies, and the generation of a large number of diverse memory B cells to help jumpstart antibody responses to subsequent infections by related pathogens. Individuals with selective defects in the GC response exhibit a range of susceptibilities to various infections. Studies of such individuals have greatly informed our understanding of the genes and molecular pathways that are important for the GC response. Recent studies have begun to determine how innate immune pathways may enhance the GC response, which may have important applications in the development of new vaccines against challenging targets. In addition, some autoimmune diseases likely involve GC reactions to produce the autoantibodies responsible for disease pathology, so increased understanding of the molecular mechanisms underlying GC responses may inform therapeutic efforts for those diseases.
